# Development of Sensory, Motor and Behavioral Deficits in the Murine Model of Sanfilippo Syndrome Type B

**DOI:** 10.1371/journal.pone.0000772

**Published:** 2007-08-22

**Authors:** Coy D. Heldermon, Anne K. Hennig, Kevin K. Ohlemiller, Judith M. Ogilvie, Erik D. Herzog, Annalisa Breidenbach, Carole Vogler, David F. Wozniak, Mark S. Sands

**Affiliations:** 1 Washington University in St. Louis, School of Medicine, St. Louis, Missouri, United States of America; 2 Saint Louis University, Department of Biology, St. Louis, Missouri, United States of America; 3 Washington University in St. Louis, Department of Biology, St. Louis, Missouri, United States of America; 4 Saint Louis University School of Medicine, St. Louis, Missouri, United States of America; 5 Washington University in St. Louis, Department of Psychiatry, St. Louis, Missouri, United States of America; Freie Universitaet Berlin, Germany

## Abstract

**Background:**

Mucopolysaccharidosis (MPS) IIIB (Sanfilippo Syndrome type B) is caused by a deficiency in the lysosomal enzyme N-acetyl-glucosaminidase (Naglu). Children with MPS IIIB develop disturbances of sleep, activity levels, coordination, vision, hearing, and mental functioning culminating in early death. The murine model of MPS IIIB demonstrates lysosomal distention in multiple tissues, a shortened life span, and behavioral changes.

**Principal Findings:**

To more thoroughly assess MPS IIIB in mice, alterations in circadian rhythm, activity level, motor function, vision, and hearing were tested. The suprachiasmatic nucleus (SCN) developed pathologic changes and locomotor analysis showed that MPS IIIB mice start their daily activity later and have a lower proportion of activity during the night than wild-type controls. Rotarod assessment of motor function revealed a progressive inability to coordinate movement in a rocking paradigm. Purkinje cell counts were significantly reduced in the MPS IIIB animals compared to age matched controls. By electroretinography (ERG), MPS IIIB mice had a progressive decrease in the amplitude of the dark-adapted b-wave response. Corresponding pathology revealed shortening of the outer segments, thinning of the outer nuclear layer, and inclusions in the retinal pigmented epithelium. Auditory-evoked brainstem responses (ABR) demonstrated progressive hearing deficits consistent with the observed loss of hair cells in the inner ear and histologic abnormalities in the middle ear.

**Conclusions/Significance:**

The mouse model of MPS IIIB has several quantifiable phenotypic alterations and is similar to the human disease. These physiologic and histologic changes provide insights into the progression of this disease and will serve as important parameters when evaluating various therapies.

## Introduction

Mucopolysaccharidosis III (MPS III, Sanfilippo Syndrome) is a lysosomal storage disease that results from a deficiency in any of four lysosomal enzymes required for the complete degradation of proteoglycans containing heparan sulfate. There are four subtypes of Sanfilippo disease: type A – sulfamidase deficient; type B – α-N-acetylglucosaminidase deficient; type C – acetyl-CoA: α-N-glucosaminide transferase deficient; type D – glucosamine-6-sulfatase deficient. Affected children appear normal in the first few years of life, but become aggressive and hyperactive [1], [2], develop sleep disturbances [3], [4] and have severe developmental delays as they age. Eventually, they regress mentally and develop loss of hearing [5], vision [6], [7] and balance. The children lose social interaction, become unable to ambulate and die of respiratory complications, heart failure or infection.

Characteristic pathologic changes are seen in the brain and other organs of MPS III patients, including lysosomal inclusions, cerebellar atrophy with loss of Purkinje cells [8], [9], [10], cortical and corpus callosum atrophy with white matter changes [11], [5], retinal pigmented epithelium (RPE) pigmentation loss, and photoreceptor degeneration [7], [12]. Usually symptom progression occurs over a decade starting around age 3–5. However, indolent adult onsets and rapidly progressive early onsets have been described [13], [14], [15], [16].

Therapy for the disease currently consists of supportive care. Antibiotics are given for infections, antipsychotics or sedatives for the behavior disturbances, and physical therapy for decreased mobility.[17]

A mouse model of MPS IIIB was created by disruption of the α-N-acetylglucosaminidase gene. Histopathologically, the mouse model demonstrated accumulation of abnormal lysosomal inclusions in multiple tissues and neuronal cell loss with concomitant astrocyte activation [18], [19]. The accumulation of heparan sulfate and gangliosides GM2 and GM3 in visceral organs and the brain was also demonstrated [20]. Clinical correlates of the histopathologic abnormalities included decreased life span, and changes in anxiety response and activity using open field testing. Interestingly, two different groups have assessed activity level using open field tests and demonstrated opposite trends [20], [21]. These groups also assessed anxiety and obtained slightly different results with different assays. The affect of vision and hearing loss on these tests was not determined.

We performed a battery of longitudinal tests of coordination, circadian rhythm, hearing, and vision, and correlated these tests with histologic findings. We describe the progression of MPS IIIB phenotypic effects in the mouse and demonstrate that the MPS IIIB mouse model has similar behavioral and sensory deficits to human MPS IIIB. The MPS IIIB mouse should serve as a reasonable model for evaluating therapeutic interventions.

## Materials and Methods

### Animals

The pedigreed congenic C57BL/6 Naglu-deficient mouse strain was acquired from The Jackson Laboratories [20], and was maintained and expanded by strict sibling mating. Wild type (+/+), heterozygous (+/−), and mutant (−/−) genotypes were determined by PCR of Naglu exon 6 and the neomycin insertion or by Naglu-4-methylumbelliferone enzyme assay [22] from tissue samples from newborn mice. Sibling matings were mutant (−/−) or heterozygous (+/−) males crossed with heterozygous (+/−) females. All animal procedures were performed in accordance with guidelines established by the Institutional Animal Care and Use Committee at Washington University in St. Louis.

### Rotarod assessment of coordination

Heterozygous and mutant mice were tested in two cohorts starting from 31 days of age (n = 18 heterozygotes and n = 16 MPS IIIB) and 217 days of age (n = 7 heterozygotes and n = 10 MPS IIIB) every 3–4 weeks for 150 days. Three paradigms were used: 1. Accelerating - acceleration over the first minute from 5 rpm to 30 rpm and maintenance of 30 rpm for the remaining 2 minutes; 2. Constant speed – continuous speed of 30 rpm for 3 minutes; 3. Rocking - reversal of direction of rotation with each full turn of the rod at 10 rpm for 3 minutes. Entrainment was done for 2 consecutive days with 3 attempts at each paradigm on a Rotarod (UGO Basile, rota-rod for mice, Varese, Italy). The first testing occurred on the third consecutive day. The length of time each mouse remained on the rod was recorded for each of the 3 attempts at each of the three paradigms. Subsequent testing was done at 3–4 week intervals with no subsequent entrainment. Mice were timed for how long they could remain on the rod for each of 3 trials under each of the 3 conditions. The longest time that each mouse remained on the rod for each condition served as the dependent variable. The data were subjected to a repeated measures ANOVA model that included one between-subjects variable (Genotype) and one within-subjects variable (Ages at testing), as well as the Huynh-Feldt adjustment of *p* values for repeated measures variables containing more than two levels. Bonferroni correction was used following multiple pairwise comparisons.

### Cerebellar Purkinje cell assessment

Cerebella from mice at ages 30 days (mutant n = 6, heterozygote n = 4) and 250–350 days (mutant n = 6, heterozygous n = 5) were obtained and immersion fixed in 4% paraformaldehyde in PBS followed by 10% neutral buffered formalin. Each cerebellum was bisected in the sagittal plane 1 micron from the midline and embedded in paraffin. Five-micron-thick sagittal sections were hematoxyline & eosin stained and Purkinje cells for each lobe of the cerebellum were counted from the mid-sagittal sections at the vermis by light microscopy. Purkinje cells were only counted if they possessed a well defined cytoplasm, nucleus and nucleolus. The number of Purkinje cells was recorded for each of the 10 lobules of each mouse cerebellum mid-sagittal section as previously delineated [23]. The cell count data were also analyzed using a repeated measures ANOVA where the model contained two between-subjects variables (genotype and age), and one within-subjects variable (lobe) followed by pairwise comparisons and other relevant contrasts.

### Circadian rhythm testing

Male mice (n = 8 wild-type, n = 9 MPS IIIB) from 70–250 days of age were individually housed in cages outfitted with a running wheel in light-tight ventilated chambers illuminated internally by fluorescent bulbs (F30T12-SP41-RS, General Electric, USA, 3.9×10^17^ to 6.9×10^18^ photons/s/m^2^ at the bottom of the cages). We recorded wheel running activity in 1 min bins (Clocklab, Actimetrics, Evanston, IL) while mice were exposed sequentially to a light-dark schedule (LD; lights on at 7:00 a.m. and off at 7:00 p.m.) for 7–14 days followed by constant darkness (DD) for 7–14 days and then a skeleton photoperiod (SK) of 1 h of light per day (lights on 1 p.m. and off at 2 p.m.) for 7 days. This sequence of LD, DD and SK was repeated 5 times to examine the ontogeny of circadian rhythms in mice with and without Naglu. Animals were given fresh water and food weekly and a clean cage after 11 days in DD.

Wheel running records were analyzed for seven parameters: period, cycle-to-cycle (day to day) period variation, phase angle of entrainment (delay between daily light offset and onset of activity), rhythm amplitude (difference from average highest to average lowest activity level), total daily activity, proportion of daily activity in the light phase of the photocycle, and duration of daily activity. Clocklab was used to determine the onset for each circadian cycle as described previously [24]. Statistical comparisons were made by 2-way ANOVA in Origin (OriginLab Corp., Northampton, MA).

### Suprachiasmatic nuclei (SCN)

Whole brains from MPS IIIB (n = 3) and heterozygous (n = 3) mice from ages 215–353 days were obtained and immersion fixed with 2% glutaraldehyde/4% paraformaldehyde in PBS. After paraffin embedding, one half- to one-micron-thick coronal slices through the region of the SCN were stained with toluidine blue and examined by light microscopy

### Auditory-evoked brainstem response (ABR) recording

Hearing in wild type and MPS IIIB mice of mixed gender (n = 7–10 of each genotype at each time point; 72 WT and 55 MPS IIIB tested in all) were examined by ABR at 4, 9, 12.5, 16.5, 20.5, 30, 36 and 45 wks of age using Tucker-Davis Technologies System 3 Complete ABR/OAE Workstation equipped with a flashlamp system and Medusa RA16 preamplifier and headstage, and a Hewlett-Packard PC computer running SigGen and BioSig software (Tucker-Davis Technologies, Alachua FL). Mice were anesthetized (80 mg/kg ketamine, 15 mg/kg xylazine, intra-peritoneal) and positioned dorsally in a custom head holder. Core temperature was maintained at 37.5±1.0°C using a thermostatically-controlled heating pad controlled by a rectal temperature probe (FHC Inc., Bowdoinham, ME). Platinum needle electrodes (Grass Technologies, West Warwick, RI) were inserted subcutaneously just behind the test ear (recording), at the vertex (reference), and in the back (ground). Responses were differentially amplified and digitally filtered using BIOSIG open source software running on a PC. Sine wave stimuli of 5, 10, 20, 40 or 56.6 kHz, having a 5 ms total duration, including 0.5 ms rise/fall times were generated digitally using SIGGEN in combination with an AP2 microprocessor, and converted using a 200 kHz D/A rate. Stimuli were presented using an ES1 electrostatic speaker located 7 cm directly lateral to either ear, concentric with the external auditory meatus. Stimuli were presented free field and calibrated using an ACO Pacific 7016¼ inch microphone placed where the external auditory meatus would normally be. Stimuli at each frequency and level were presented 1,000 times at 20/sec. The minimum sound pressure level required for detection of a response was determined using a 5 dB minimum step size. The mean response, standard deviation, and standard error of the mean were calculated for each group of genotype and age-matched animals. Statistical analysis, consisting of two-way ANOVA followed by Tukey pairwise multiple comparisons, were performed using Sigma-Stat software (SPSS, Chicago, IL).

### Middle & inner ear histopathology

Because optimal preservation of middle versus inner ear involves incompatible procedures, these were examined in different animals, using somewhat different methods. To obtain middle ear samples, five each of 30 wk old wild type and MPS IIIB mice of mixed gender were sacrificed by overdose with pentobarbital and decapitated. Intact middle and inner ears were then dissected from the skull as a single piece connected by the dorsal plate of the skull, and immediately immersed in cold 4% paraformaldehyde/2% glutaraldehyde in PBS. A small hole was then made in each auditory bulla to promote permeation of dehydration and embedding media. Samples were fixed greater than 24 hours, post-fixed in buffered 1% osmium tetroxide, decalcified for 30 days in 0.35 M sodium EDTA, dehydrated in an ascending acetone series, and embedded in Epon. Left and right bullas were then separated, sectioned in the horizontal plane at 4.0 µm, and stained using toluidine blue.

To examine inner ears, five each of 30 wk old WT and MPS IIIB mice of mixed gender were overdosed with pentobarbital and decapitated. Each cochlea was rapidly isolated, immersed in cold 2.0% paraformaldehyde/2.5% glutaraldehyde in 0.1 M phosphate buffer (pH 7.4), and the stapes was removed. Samples were placed in the same fixative for greater than 24 hours, and decalcified in sodium EDTA for 72 hours. Next they were post-fixed in buffered 1% osmium tetroxide, dehydrated in an ascending acetone series, and embedded in Epon. Cochleae were sectioned in the mid-modiolar plane at 4.0 µm (permitting assessment of both cochlear duct and vestibular organs), then stained with toluidine blue. Images taken for illustration were captured using a Diagnostic Instruments Model 1.4.0 digital camera controlled by Openlab™ software, and further processed using Canvas™.

A minimum of 50 sections were obtained from one or both middle/inner ears from each animal for bright field viewing with a Nikon Optiphot™ light microscope. Middle ears and cochleae, along with vestibular organs, were examined qualitatively. To confirm an impression of loss of cochlear hair cells and afferent neurons in the cochlear base of MPS IIIB mutant mice, 5 mid-modiolar cochlear sections (spaced 5 sections apart) from 5 WT and 5 mutant mice were also assessed quantitatively. To estimate hair cell loss, the number of complete or partial hair cell profiles in the organ of Corti (as viewed in radial section) was averaged across 5 sections in each animal. To estimate neuronal loss, nucleated profiles within the spiral ganglion were counted within a 3,600 µm^2^ area using a calibrated grid ocular, centered over Rosenthal's canal, averaged over 5 sections.

### Electroretinography

Flash electroretinography was performed as described previously on wild type and MPS IIIB mice of mixed gender [25], [26] at ages 4, 8.5, 12.5, 16, 20.5, 30, and 40 weeks (n = 7–10 of each genotype at each time point; 58 WT and 58 MPS IIIB tested in all). The mice were dark-adapted for a minimum of 2 hours to obtain a mixed rod/cone ERG. Mice were anesthetized (80 mg/kg ketamine, 15 mg/kg xylazine, intra-peritoneal) and positioned dorsally in a custom head holder. Core temperature was maintained at 37.5±1.0°C using a thermostatically-controlled heating pad controlled by a rectal temperature probe (FHC Inc., Bowdoinham, ME). The recording electrode was placed in a drop of atropine/methylcellulose on the surface of the cornea of the eye, the reference electrode was inserted subcutaneously at the midline of the cranium, and a ground electrode was placed subcutaneously in the back. Mice were exposed to light flashes of 10 ms duration and an intensity of 76.2 cd*s/m^2^. Flash ERG measurements were recorded on a Tucker-Davis System 3 Complete ABR/OAE Workstation (Tucker-Davis Technologies, Alachua, FL, USA) and analyzed using BioSig software. Dark-adapted mixed rod/cone ERG measurements were determined from the average response to 5–10 presentations of light at 0.1 Hz. For light-adapted pure cone ERG measurements, mice were allowed to adapt to light with an intensity of 72.7 cd/m^2^ for 20 min. ERG measurements were obtained by measuring the average response following 50 light flashes at a frequency of 1 Hz. Comparisons were made of the b-wave amplitudes in microvolts (most negative to most positive points in the trace) from the best of three measurements of each of the light-adapted and dark-adapted responses, and then averages were determined for each group of animals. The mean response, standard deviation, and standard error of the mean were calculated for each group of genotype and age-matched animals. Statistical analysis, consisting of two-way ANOVA followed by Tukey pairwise multiple comparison, was performed using Sigma-Stat software (SPSS, Chicago, IL).

### Retinal histology

Eyes were obtained from wild type (n = 1–6) and MPS IIIB (n = 1–6) mice at 4, 8, 12, 16, 20, 30, 34, and 45 weeks of age and immersion fixed in 2% glutaraldehyde/4% formaldehyde in PBS. Samples were embedded in Epon-Araldite resin and sectioned into 1-micron-thick sections prior to staining with toluidine blue. Imaging was obtained by light microscopy.

## Results

### Motor function & Purkinje cell counts

**Figure 1:**
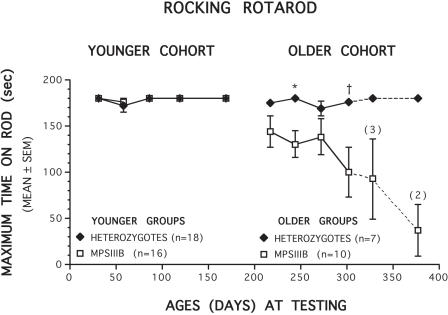
Older MPS IIIB mice are impaired on the rocking rotarod. Mean duration (in seconds) of the time spent on the rocking rotarod as a function of increasing ages (days) for MPS IIIB (open squares) and heterozygous (filled diamonds) control mice. No performance differences were observed between groups on the rocking rotarod in the younger cohort of mice (left panel). In contrast, an ANOVA conducted on the first 4 data points from the older cohort of mice (right panel) revealed a significant main effect of Genotype (p = 0.030), thus showing, in general, that the older MPS IIIB mice, remained on the rod for a significantly shorter time compared to the heterozygous control mice. Differences between groups were greatest when the mice were tested at 244 (*p = 0.013) and 302 (†p = 0.034) days of age. Even greater deficits were observed in the MPS IIIB mice when they were tested at older ages although small sample sizes resulting from high mortality rates in the MPS IIIB mice precluded formal statistical analyses. Numbers in parentheses represent sample sizes at specific ages of testing. The dotted line indicates that the last two test sessions (327 and 372 days) were not included in the overall ANOVA since sample sizes were so small due to increased mortality.

No performance deficits were observed in the MPS IIIB mice in either the younger or older cohorts on the common rotarod measures involving the constant speed or accelerating conditions across the ages of testing (data not shown). We had included the rocking rotarod condition in our protocol in an effort to enhance test sensitivity by increasing the difficulty of the task as a result of the direction reversal with each rotation of the rod. Use of the rocking rotarod did not produce performance differences between groups in the young cohort of mice where the MPS IIIB and control mice performed almost identically across the different ages of testing (Fig. 1). However, testing the mice on the rocking rotarod did lead to performance deficits in the MPS IIIB mice in the older cohort (Fig. 1). An ANOVA was conducted on the data from the first four test sessions in the older cohort of mice since we wanted to assess performance before significant mortality began to occur. The ANOVA yielded a significant main effect of Genotype, [F (1, 15) = 5.75, p = 0.030], and subsequent pairwise comparisons showed differences were greatest at the second (p = 0.013) and fourth (p = 0.034) test sessions when the mice were 244 and 302 days old, respectively. This is well before the average age of death for the MPS IIIB animals at 315–360 days. Even greater differences were observed during the fifth and sixth test sessions, although significant attrition through mortality had occurred by this time making the data insufficient for legitimate analyses.

**Figure 2:**
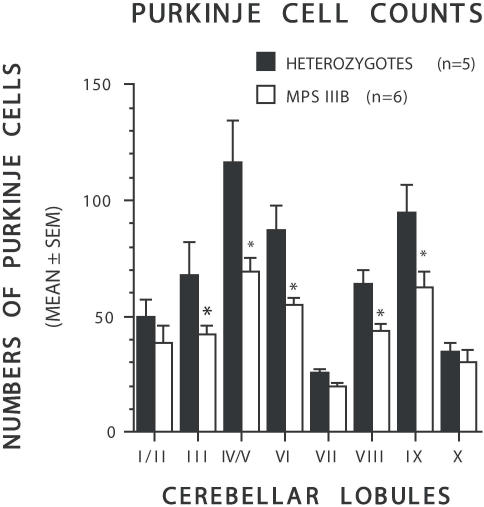
Age-related progressive loss of Purkinje cells in the cerebellar lobules in MPS IIIB mice. ANOVA of Purkinje cell count by lobe showed that old MPS IIIB mice (white bars) had significantly lower neuronal counts across cerebellar lobes compared to old heterozygous controls (black bars) although differences between groups were lobe dependent. Pairwise comparisons conducted in the older cohort of mice showed that differences between groups were greatest in lobes VI (*p = 0.001), IV/V (*p = 0.003), VIII (*p = 0.004), IX (*p = 0.011), and III (*p = 0.036). An ANOVA of the Purkinje cell count data that included both young and old cohorts of mice resulted in a significant main effect of Age (p<0.001) and a significant Age by Genotype interaction (p = 0.003). Other important effects included significant Lobe by Genotype (p = 0.010), and Lobe by Age (p<0.001) interactions. Additional comparisons showed that the older MPS IIIB mice had significantly fewer neurons across the cerebellar lobes compared to the younger MPS IIIB mice (p<0.001, (p<0.006, the Bonferroni corrected value)), while no differences were found between older and younger control mice.

In general correspondence with the age-related impaired performance on the rocking rotarod test of MPS IIIB mice was the finding of a diffuse decrease in cerebellar Purkinje neuronal counts at the vermis (Fig. 2) in older MPS IIIB mice but not in younger MPS IIIB mice. This age-dependent loss of Purkinje cells was documented by the results of an ANOVA which yielded a significant main effect of Age, [F (1,16) = 31.15, p<0.001], and a significant Age by Genotype interaction, [F (1,16) = 12.57, p = 0.003]. Other results included a significant effect of Lobe, [F (7,112) = 109.98, p<0.001], a significant Lobe by Genotype interaction, [F (7,112) = 3.00, p = 0.010], and a significant Lobe by Age interaction, [F (7,112 = 9.53, p<0.001]. Subsequent contrasts showed that old MPS IIIB mice had, in general, significantly lower neuronal counts across the cerebellar lobes compared to their older counterpart controls, [F(1,16) = 15.29, p = 0.001], while neuronal counts across cerebellar lobes were not different between young MPS IIIB mice and young controls. Pairwise comparisons conducted in the older cohort of mice showed that differences between groups were greatest in lobes VI (p = 0.001), IV/V (p = 0.003), VIII (p = 0.004), IX (p = 0.011), and III (p = 0.036). To further clarify the age-related Purkinje cell loss, we compared neuronal counts between older and younger MPS IIIB mice and found that, in general, the older MPS IIIB mice had significantly fewer neurons across the cerebellar lobes compared to the younger MPS IIIB mice, [F(1,16) = 46.38, p<0.001], while no differences were found between older and younger control mice.

### Circadian locomotion & SCN histology

**Figure 3:**
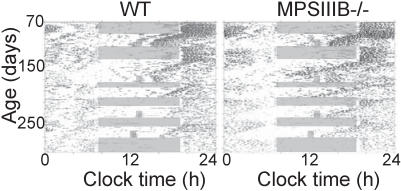
Locomotor activity records of a representative wild-type and MPS IIIB mouse. Running wheel revolutions per minute (black bars) is plotted for 24 h on each line with subsequent days plotted on the line below. In a light:dark schedule (gray bars show when lights were on), the mutant mice adjusted rhythm to light similar to wild type mice. The mice experienced a series of light:dark, dark:dark, and skeleton photoperiod schedules from ages 70–275 days.

**Figure 4:**
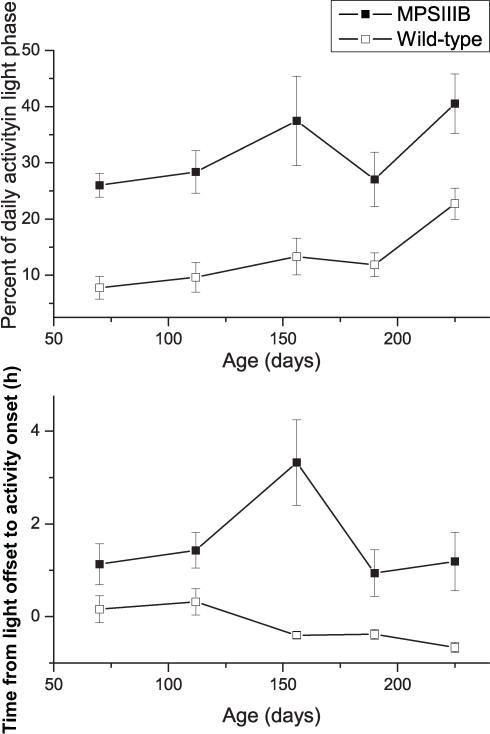
Daily activity in light phase (%) and time (hours) to onset of activity after light offset. MPS IIIB mice had a greater percentage of total activity performed during light phases than the WT mice. MPS IIIB mice also had a longer phase angle of entrainment with a delay in starting daily activity after lights were turned off of >1 hour. Both alterations were present from ages 70 to 225 days.

Long-term recordings from MPS IIIB and wild-type mice from 70–250 days of age showed that they both entrain to light-dark and skeleton light cycles and free-run in constant darkness (Fig. 3). However, MPS IIIB mice consistently differed from wild-type mice at all ages examined in their phase angle of entrainment (the time from the light offset to the onset of daily locomotor activity) (Fig. 4). MPS IIIB mice started their daily activity approximately 1 h later than wild-type mice (p<0.00001 for the effect of genotype, but not significant for the effect of age by post hoc analyses). The percentage of daily activity which occurred during the light portion of the LD cycle was increased in the MPS IIIB mice compared to the wild-type mice (2-way ANOVA, p<0.00001 for the effect of genotype; not significant for the effect of age by post hoc analyses except at the oldest age tested). MPS IIIB mice did not differ significantly from wild-type mice in period, cycle-to-cycle period variation, rhythm amplitude, total daily activity, and duration of daily activity.

**Figure 5:**
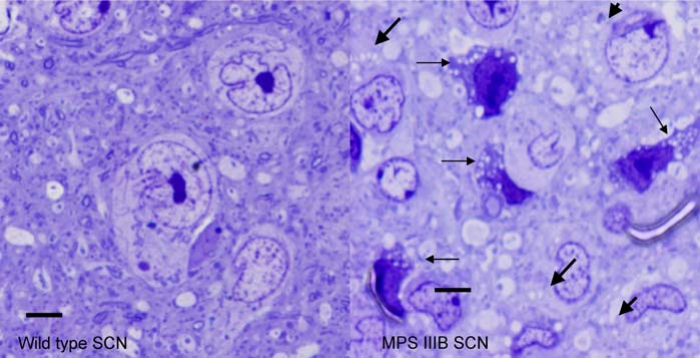
Suprachiasmatic nuclei (SCN) from wild type and MPS IIIB mice. The SCN of wild type mice contains few if any pyknotic cells with no observed lysosomal distention. MPS IIIB SCN has numerous pyknotic cells indicated with small black arrows and non-pyknotic cells with distended lysosomal inclusions indicated by large arrows. Bars indicate 10 micrometers.

In mammals, the suprachiasmatic nucleus (SCN) of the hypothalamus drives daily rhythms in locomotor activity [27]. Long-term recordings of wheel running in rodents provides a real-time, non-invasive measure of the state of the SCN, inputs to the SCN and the ability to synchronize to a light-dark schedule and control daily rhythms in locomotion. In the SCN of MPS IIIB mice there are a large number of pyknotic cells (with condensed nuclei and cytoplasms) suggesting an increase in apoptotic and/or degenerating neurons. The pyknotic cells contain numerous distended lysosomes. The majority of neurons in the mutants that are not pyknotic also demonstrate lysosomal distention, whereas none of the neurons in the wild type SCN contain inclusions. (Fig. 5)

### Auditory function & middle and inner ear histopathology

**Figure 6:**
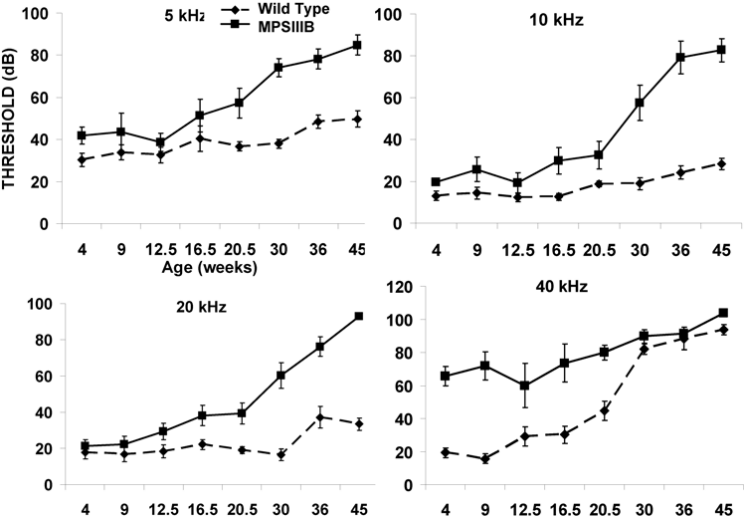
Auditory-evoked Brainstem Responses (ABR). Mean (±SEM) ABR thresholds in MPS IIIB and WT mice at 5, 10, 20 and 40 kHz from age 4 to 45 weeks. Earliest differences by genotype appear at high frequencies (40 kHz). By 30 wks, high frequency hearing losses due to background deficits in C57BL/6 dominate. Threshold differences at lower test frequencies, apparent after 16 wks, probably reflect mixed cochlear and middle ear pathology. Differences by genotype were significant at all ages (p<.001, 2-way ANOVA).

The C57BL/6 background includes alleles that promote progressive sensorineural hearing loss [28], therefore both wild type and MPS IIIB mice show accelerated high frequency (40 kHz) ABR threshold elevation (Fig. 6). However, the increase in high frequency ABR thresholds is significantly greater in the MPS IIIB mice up to 16 wks, at which time background effects begin to dominate. The fact that initial threshold differences are manifest only at high frequencies suggests that a sensorineural component of hearing loss precedes the conductive component. Later emergence of a conductive component is suggested by the relatively ‘flat’ threshold elevation (i.e., vertically shifted from WT at all frequencies, but similar audiogram shape) at lower stimulus frequencies. Presumably both sensorineural and conductive effects influence thresholds at most frequencies by 16 wks.

**Figure 7:**
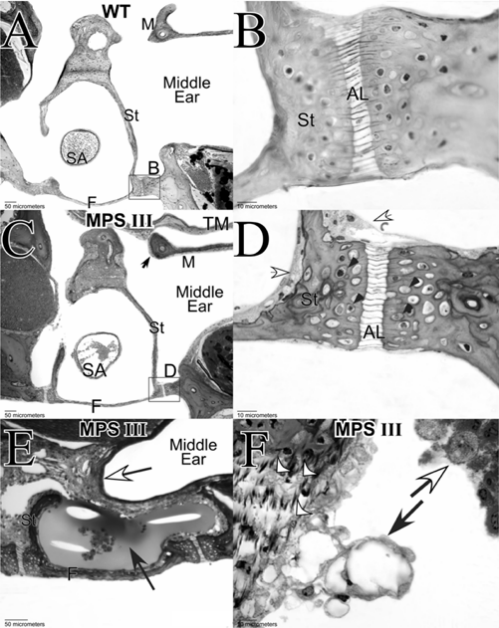
Middle ear pathology. A. Cross-section of stapes (St) with malleus (M) in WT mouse. B. Higher magnification of rectangular area in A shows thin mucosal lining and regular bone surfaces. C. Analogous view to A in an MPS IIIB mouse at age 30 weeks showing thickened mucosal layer on stapes and malleus (arrow). D. Enlarged view of rectangular area in C showing hyperplasia of mucosal cells (white arrows) and lysosomal storage in osteocytes (black arrowheads). Bone surface is pitted, indicating abnormal bone remodeling. E. Orthogonal view of stapes in a different MPS IIIB mouse showing otitis media with effusion. Note hyperplasia of mucosal cells (white arrow) and infiltrate with inflammatory cells (black arrow). F. Articulation point between incus and malleus in MPS IIIB mouse showing storage in chondrocytes (white arrowheads), hyperplasia and storage in mucosal cells (black arrow), and storage in inflammatory cells (white arrow). SA: Stapedial artery; F: Footplate of stapes; AL: Annular ligament at point of stapes insertion into cochlea; TM: Tympanic membrane.

Middle ears from MPS IIIB mice at 30 wks of age showed a combination of storage-related anomalies and highly variable otitis media, with and without effusion (Fig. 7). Lysosomal storage was prominent in the mucosal lining and within osteocytes, chondrocytes, and inflammatory cells (when present). Ossicles were covered by an extended and thickened mucosal layer (Fig. 7C, D). Bony surfaces were rough and pitted, suggesting abnormal bone remodeling. When otitis media with effusion was present (Fig. 7E, F) inflammatory cells and all cells of the hypertrophied mucosal lining showed storage. This middle ear pathology would be expected to alter the mass, stiffness, and internal damping of the middle ear, and contribute to a conductive hearing loss.

**Figure 8:**
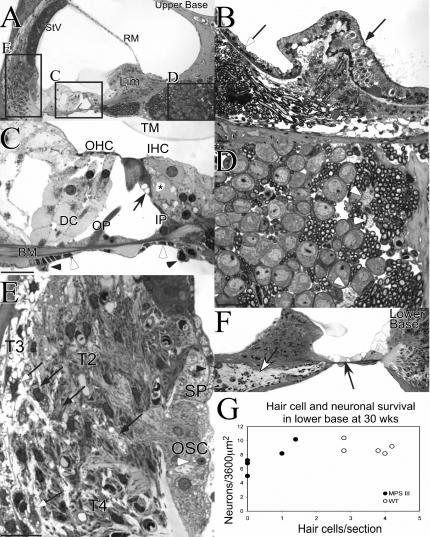
Inner ear pathology in MPS IIIB mice at 30 wks. A. Radial view of cochlear upper basal turn showing grossly normal features. B. Crista ampullaris of lateral semicircular canal in the same animal shows abnormal lysosomal storage in both hair cells and supporting cells of the sensory epithelium (black arrow), and in dark cells (white arrow). C. Expanded view of medial organ of Corti in A. The only cells showing storage are inner pillar cells (IP) (black arrow). Mesothelial cells lining the basilar membrane (BM) show the aberrant storage (white arrowheads), as do adherent inflammatory cells (black arrowheads). Vacuoles within inner hair cells (asterisk, IHC) are probably a processing artifact. D. Spiral ganglion cell region from A shows aberrant storage only in glial cells (white arrowheads). E. Spiral ligament and lateral organ of Corti from A shows aberrant storage in outer sulcus cells (white arrowhead, OSC), epithelial cells of spiral prominence (black arrowhead, SP), Type III fibrocytes (white arrows, T3), and Type II fibrocytes (black arrows, T2). Type IV fibrocytes (T4) also showed storage, not apparent in this view. F. Complete loss of hair cells and other differentiated cell types of the organ of Corti (black arrow), with secondary loss of neuronal processes (white arrow). This was more prevalent in MPS IIIB mice than in WTs. G. Plot of the number of hair cell profiles versus neuronal density, as seen in radial view in the lower base for 5 MPS IIIB and 5 WT mice at 30 wks. Differences in hair cell numbers by genotype are highly significant (t-test, p<.001). Because inner hair cells were less affected than outer hair cells, only numbers <1.0 indicate loss of IHCs. Neuronal density decreases only when IHCs are missing, so that neuronal loss appears secondary to hair cell loss in MPS IIIB. StV: Stria vascularis; RM: Reissner's membrane; TM: Tectorial membrane; OHC: Outer hair cells; DC: Deiters' cells; OP: Outer pillar cell; Lim: Spiral limbus.

Inner ear pathology encompassed a host of cell types in the cochlea. However, the organ of Corti was largely spared (Fig. 8). Storage was evident in the spiral ligament (Fig. 8E) (Type II, III, and IV fibrocytes, and epithelial cells of the spiral prominence), the spiral limbus (stellate fibrocytes and interdental cells), Reissner's membrane (both epithelial and mesothelial cells), and mesothelial cells lining the bony capsule. No storage was observed within the stria vascularis. Although spiral ganglion cells did not show storage, adjacent glial cells frequently did (Fig. 8D). The only storage noted in the organ of Corti occurred within outer sulcus cells of the lateral organ (white arrowheads in Fig. 8E) and pillar cells of the medial organ (black arrow in Fig. 8C). Inflammatory cells were more likely to appear in the perilymphatic scalae of mutant mice than wild types (black arrowheads in Fig. 8C), and often showed storage. By contrast with the organ of Corti, the vestibular maculae and cristae showed prominent storage in both supporting cells and hair cells (Fig. 8B), as did dark cells of the cristae (analogous to cochlear stria marginal cells).

Extensive lysosomal storage throughout the inner ear did not generally appear to promote degeneration. Nevertheless, our material yielded an impression of marked hair cell and neuronal loss in the cochlear lower basal turn in MPS IIIB mice. Since this can also occur due to aging in C57BL/6 mice, mutant and wild type mice were compared quantitatively. Figure 8G plots hair cell profiles/radial section versus neuronal density in the lower base for MPS IIIB and wild type mice at 30 wks of age. Despite the small sample, there is no overlap of hair cell numbers by genotype and the difference is significant (t-test, p<0.001). Without exception, outer hair cell loss was more pronounced than inner hair cell loss, so that numbers <1.0 exclusively indicate loss of inner hair cells. The data indicate that neuronal density decreases only when all hair cells, including inner hair cells, are lost. Since the vast majority of spiral ganglion cells innervate inner hair cells, this suggests that neuronal loss in MPS IIIB occurs secondary to loss of inner hair cell targets, and that MPS IIIB does not directly promote neuronal loss. Complete loss of hair cells in the lower base tended to coincide with wholesale degeneration of the organ of Corti (Fig. 8F). While this can also be found in comparably aged C57BL/6 mice, it was much more pronounced in the MPS IIIB mice.

### Retinal function & histology

**Figure 9:**
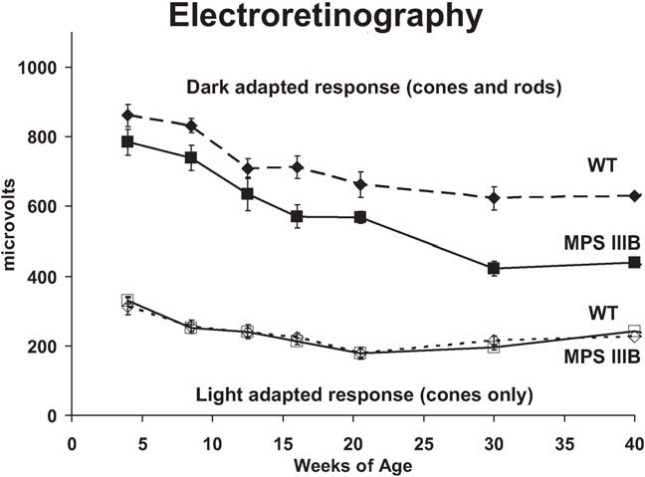
Electroretinography. Mixed (cones and rods) photoreceptor b-wave amplitudes (microvolts) to a light stimulus in dark-adapted wild type (diamonds on dashed line) and MPS IIIB (squares on solid line) mice at ages 4 to 40 weeks demonstrating reduced response in MPS IIIB mice, that is statistically significant from 16 weeks onward. Cone response b-wave amplitudes after light adaptation is depicted for wild type (open diamond/dashed line) and MPS IIIB (open square/solid line) at the same ages and shows no difference in cone response.

The dark-adapted retinal response is depressed by 5 weeks in MPS IIIB mice and becomes progressively less sensitive with increasing age. The diminished sensitivity reflects a loss of rod function that becomes statistically significant after 15 weeks. There are no significant differences at any age in retinal function after light adaptation suggesting a normal level of cone function. (Fig. 9)

**Figure 10:**
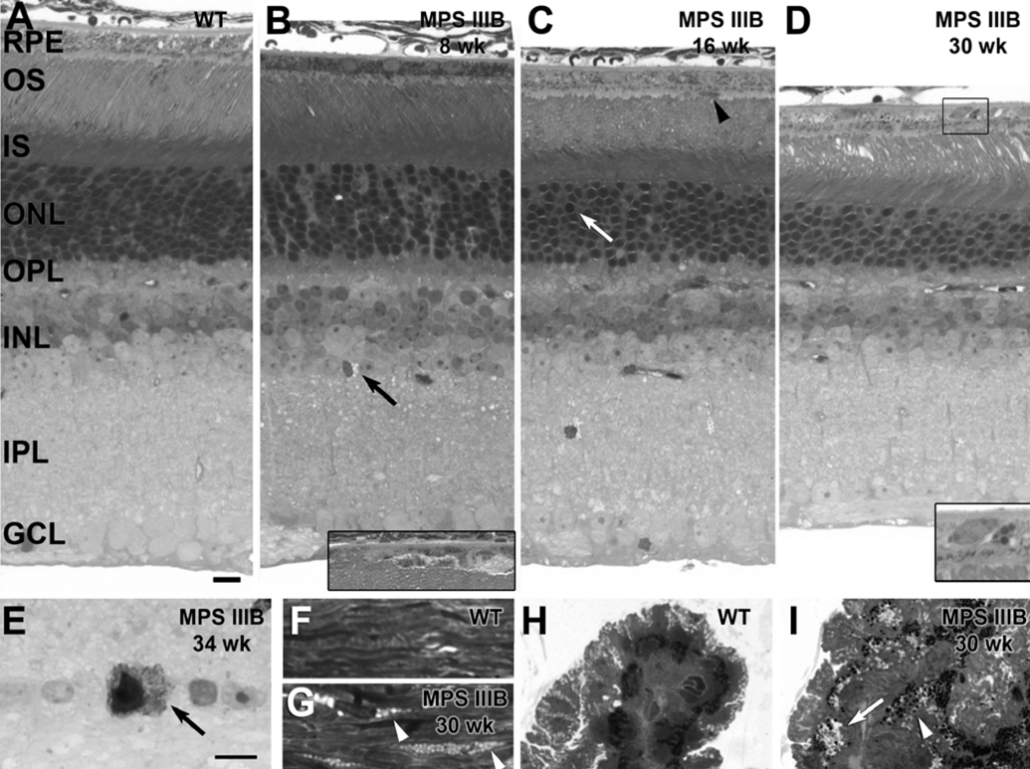
Eye Pathology. Light micrographs showing pathology from normal (A, F, H) and MPS IIIB (B–D, E, G, I) mouse retinas (A–E), sclera (F, G), and ciliary bodies (H, I). (A) Normal histology from a 4-week old wild type mouse retina is shown and is indistinguishable from older wild type retinas at 45 weeks. (B) In the 8-week old MPS IIIB mouse retina, aberrant lysosomal storage can be seen in non-neuronal cells in the inner retina (arrow). Inset: Localized disruption of the retinal pigment epithelium (RPE) from a nearby region of the same retinal section is shown. (C) At about 16 weeks, the outer segments (OS) of the MPS IIIB mouse are shortened, the outer nuclear layer (ONL) is reduced by 2–4 rows of nuclei, and pyknotic nuclei are seen in the ONL (arrow). Macrophage-like cells are present in the subretinal space (arrowhead). (D) In the 30-week old MPS IIIB mouse retina, OSs are further shortened and the ONL is reduced by nearly half. Inset: higher magnification of the boxed area showing dense, round melanosome-like structures in the RPE. (E) By 34 weeks, a subpopulation of cells in the ganglion cell layer (GCL) has a dense appearance with numerous lysosomal inclusions in the cytoplasm. (F) Normal histology from a 4-week old wild type mouse sclera is shown. (G) Lysosomal storage appears as pale vesicles in the sclera of the MPS IIIB mouse (arrowheads). (H) Normal histology from a 4-week old wild type mouse ciliary body is shown. (I) By 30 weeks of age, the ciliary body of the MPS IIIB mouse appears disorganized and swollen with lysosomal storage vesicles (arrow). Numerous dense, round melanosome-like structures are seen (arrowhead). IS, photoreceptor inner segments; OPL, outer plexiform layer; INL inner nuclear layer; IPL, inner plexiform layer. Smaller bar = 10 micron for A–D, H, I. Larger bar = 10 micron for E–G.

Histological analysis was performed on retinas from control (n = 3–6) and MPS IIIB (n = 1–6) mice at 4, 8, 12, 16, 20, 30, 34, and 45 weeks of age. At 4 weeks of age, the MPS IIIB retina appeared grossly normal, although localized abnormalities were seen in retinal pigment epithelium (RPE) cell shape consistent with loss of cell polarization and/or delamination (not shown). By 8 weeks of age, lysosomal storage could be seen in vascular cells, Muller glial cells and microglia in the inner retina of MPS IIIB mice (Fig. 10B) compared to age matched wild type controls (Fig. 10A). Occasional pyknotic nuclei could be seen in the outer nuclear layer (ONL) and further disruption in the RPE was seen. Photoreceptor degeneration, with shortening of the outer segments and cell loss in the outer nuclear layer, became apparent around 16 weeks (Fig. 10C) with a decrease of 2–4 rows of nuclei by 20 weeks of age. Macrophage-like cells were apparent in the sub-retinal space by 16 weeks. Nearly half of the photoreceptor cells were gone by 30–34 weeks (Fig. 10D) with only 3–4 rows of nuclei remaining at 45 weeks (not shown). Outer segments are further shortened and appear swollen. A small population of cells in the ganglion cell layer began to show pathology by 34 weeks (Fig. 10E). These cells were usually large and irregularly shaped, with numerous inclusions in the cytoplasm. The cytoplasm and nucleus of these cells appeared darker than other cells in the ganglion cell layer.

Large, round, dense melanosome-like structures were seen in the RPE of MPS IIIB retinas, first becoming noticeable in the periphery of the mutant eye as early as 4 weeks, distributed throughout the RPE by 16 weeks, and becoming larger and more prominent by 30 weeks (Fig. 10D, inset). While melanosomes in normal retinas were typically ovoid in shape and localized to the apical cytoplasm, these structures were prominent in the mid to basal cytoplasm and were distinguished by their round shape. Although similar small structures were seen in control eyes at 30–45 weeks, they were seen at younger ages and appeared larger and more numerous in the mutant eyes. Lysosomal storage was also seen in the sclera (Fig. 10F, G) and ciliary body (Fig. 10H, I) of MPS IIIB mice. Inclusions were first apparent by 8 weeks of age with increasing accumulation and disruption of the tissues by 30 weeks. The ciliary body included round, dense melanosome-like structures, similar to those seen in the RPE.

## Discussion

### Motor function and Purkinje cell loss

In humans with MPS IIIB, the loss of ability to walk has long been described and histological studies have revealed a loss of Purkinje cells in addition to the accumulation of lysosomal inclusions [9]. The MPS IIIB mouse, as described above, also develops difficulty with coordination as measured by the rocking rotarod technique, though the effect is not as dramatic as is seen in humans. Joint stiffness, which has been described in affected children, may contribute to changes in motor or sensorimotor functions in the MPS IIIB mice. However, the lack of performance differences between MPS IIIB and normal mice between the constant speed or accelerating conditions of the rotarod test argues against joint stiffness as a major source of coordination deficits. The significant progressive loss of Purkinje cells likely contributes to the performance deficits on the rocking rotarod, though the additional contribution of structural and neuronal deficits in the vestibular apparatus has not been ascertained. However, the absence of significant Purkinje cell deficits in the archicerebellum is consistent with a relatively normal central vestibular system. Our assessment of Purkinje cells was limited to the vermis while the cerebrocerebellum was not assessed. The decrease in neuronal counts seen near the midline in the paleocerebellum and neocerebellum would be expected to result in deficits in muscle tone and coordination. Consistent with this, MPS IIIB mice have a visibly altered posture and a wide gait, and although coordination performance deficits were only detectable under the relatively challenging conditions of the rocking rotarod procedure, they nevertheless represent a measurable motor deficit affecting coordination and/or balance. The deteriorating performance on the rocking rotarod and the progressive loss of Purkinje cells seen in the MPS IIIB mouse represent reasonable parallels with the human disease and will serve as objective measures when evaluating therapeutic interventions.

### Circadian deficits

Sanfilippo syndrome children demonstrate initial hyperactivity and behavioral disturbances with parents reporting increased nighttime activity. The children subsequently have diminished activity levels but retain the nighttime wakefulness. Measuring MPS IIIB mice using real-time locomotor monitoring during the progression of lysosomal storage, we found alterations in the timing of daily locomotor activity at the earliest ages tested. Mice are normally nocturnal with activity highest during darkness and reduced activity during the light time. The MPS IIIB mice demonstrated a significant increase in light phase activity which is consistent with the increased nighttime activity seen in affected children [3], [29], [4], [30]. The total activity level did not significantly differ between MPS IIIB and wild type mice over 70–250 days of age. A period of hyperactivity was not observed but may have been missed if it occurred prior to 3 months of age. All mice showed a normal decrease in activity with increasing age.

Our findings are in contrast to those of prior studies on MPS IIIB mice [20], [21] and further characterize activity levels. One group tested mice at 2.5 and 4.5 months and found low normal activity at 2.5 months but significantly diminished activity at 4.5 months. This contrasts with the findings of the second group, who found normal activity at 5 weeks and increased activity at 29 weeks. Both of these studies tested mice in an open field rather than a home cage running wheel so activity differences due to contextual change (novelty) cannot be assessed using our methods, although our use of longer assessment periods provides a more accurate index of mouse activity. Both groups also assessed spontaneously-occurring and conditioned anxiety-like behaviors. The first group assessed learning in a Pavlovian fear conditioning test and demonstrated diminished freezing (conditioned anxiety) in response to a tone that had been paired with shock at the older age of 4.5 months. However, no differences in freezing were observed when the mice were placed back into the chamber where they had previously received tone-shock pairings to assess contextual fear conditioning. The second group used the elevated plus-maze test to assess anxiety-like behaviors related to avoidance of open arms of a maze at 18 and 34 weeks and demonstrated reduced anxiety response at both ages. The affect of hearing and vision deficits on anxiety testing was not determined.

MPS IIIB mice showed the greatest activity level changes in the presence of a light-dark cycle. In constant darkness, the level of and duration of locomotor activity did not differ between genotypes. In a light-dark cycle, the fragmented activity during the light phase and delayed activity onset (phase angle of entrainment) during the dark phase of MPS IIIB mice indicate that the disease preferentially targets the pathways responsible for light responsiveness, with little effect on the mechanisms responsible for circadian rhythm generation in the SCN. This is despite the increased numbers of pyknotic neurons and marked accumulation of inclusions in the cells in the SCN. The preservation of circadian function despite obvious SCN pathology supports previous observations that only a small portion of the SCN suffices to coordinate daily rhythms in locomotion [31]. That the MPS IIIB mice also become active later than wild-type mice may indicate deficits in the output pathways from the SCN to brain areas involved in waking behaviors.

### Auditory deficits

Middle and inner ear pathology in MPS III are not well described in humans or animals. Hearing loss typically appears mixed, while clear indications of vestibular deficits are not reliably observed [32], [33], [34]. Many cochlear cell types showed aberrant lysosomal storage in MPS IIIB mice, including outer sulcus cells and pillar cells of the organ of Corti, most fibrocytes of the spiral ligament, and fibrocytes and interdental cells of the spiral limbus. The wide variety of cochlear cells affected could alter fluid homeostasis and the driving force for receptor currents. Although limited lysosomal storage is found in the organ of Corti in MPS IIIB (none in hair cells), abnormal lysosomal storage within mesothelial cells lining the basilar membrane could alter passive mechanics which could elevate hearing thresholds. MPS IIIB mice feature marked lysosomal storage within receptor and supporting cells of all the vestibular organs. It remains unclear whether this impacts vestibular function.

The MPS IIIB mouse demonstrates hair cell loss and organ of Corti degeneration in the lower cochlear base. If this finding extends to humans, it holds implications for eventual therapies, in that such degenerative changes are not reversible. In contrast, non-degenerative aspects of MPS-related inner ear pathology appear largely reversible [35], [36]. It may also be the case, however, that abnormal bone remodeling in the middle ear [probably common to several MPS forms [37], [38]] cannot be completely reversed. The sensorineural hearing loss observed with MPS IIIB humans [5] is reproduced in the disease phenotype of the MPS IIIB mouse though the correlative anatomic studies in humans have not been documented.

### Retinal Function

Electroretinography performed in MPS IIIB mice demonstrates a loss of rod function that correlates with diffuse retinal pathology including photoreceptor degeneration and vacuolar inclusions in the retinal pigmented epithelium. Multiple studies of Sanfilippo children have shown similar abnormalities: diminished to absent vision with abnormal ERGs [6], photoreceptor degeneration, and RPE inclusions [7]. In addition, the Sanfilippo children have inclusions in the retinal ganglion cells, ciliary body and sclera, also observed in the MPS IIIB mouse eye [7]. Pathologic changes in the ganglion cell layer are particularly interesting in view of the observed circadian deficits, since ganglion cells rather than photoreceptors are the primary input to the circadian system [39]. Inclusions in the lens epithelium, cornea, iris, and trabecular meshwork are also seen in Sanfilippo children, though we did not assess for inclusions in these regions of the mouse eye. No mention is made in the human studies of changes in the thickness of the outer nuclear layer and outer segments, though these changes may have been more noticeable in our mice studies due to direct comparison with normal eyes [6], [7], [12].

### Summary

We demonstrate that the murine model of MPS IIIB develops progressive deterioration of balance, vision, and hearing. We show that the development of histological changes in the MPS IIIB mouse correlates with the functional changes in balance and vision. We establish that the murine model of MPS IIIB has similar phenotypic manifestations to those seen in the human disease with: altered balance and loss of cerebellar neurons; abnormal sleep patterns and imbalanced day to night activity level; hearing loss; diminished vision and typical inclusions in the RPE. The present study describes the progression of phenotypic changes in the MPS IIIB mouse and sets useful parameters for future assessment of therapies with the mouse model that are reflective of the human disease.
